# Engaging physicians and nurses in balanced scorecard evaluation—An implication at Palestinian hospitals and recommendations for policy makers

**DOI:** 10.3389/fpubh.2023.1115403

**Published:** 2023-03-07

**Authors:** Faten Amer, Arie Arizandi Kurnianto, Abdulsalam Alkaiyat, Dóra Endrei, Imre Boncz

**Affiliations:** ^1^Doctoral School of Health Sciences, Faculty of Health Sciences, University of Pécs, Pécs, Hungary; ^2^Institute for Health Insurance, Faculty of Health Sciences, University of Pécs, Pécs, Hungary; ^3^School of Pharmacy, Faculty of Medicine and Health Sciences, An-Najah National University, Nablus, Palestine; ^4^Division of Public Health, Faculty of Medicine and Health Sciences, An-Najah National University, Nablus, Palestine; ^5^National Laboratory for Human Reproduction, University of Pécs, Pécs, Hungary

**Keywords:** attitude of health personnel, balanced scorecard, delivery of health care, health services administration, hospital administration, quality of health care

## Abstract

**Introduction:**

Healthcare workers (HCWs) are seldom involved in balanced scorecard (BSC) deployments. This study aims to incorporate Palestinian HCWs in the BSC to create health policy recommendations and action plans using BSC-HCW1, a survey designed and validated based on BSC dimensions.

**Methodology:**

In this cross-sectional study, the BSC-HCW1 survey was delivered to HCWs in 14 hospitals from January to October 2021 to get them involved in PE. The differences between physicians' and nurses' evaluations were assessed by the Mann–Whitney *U*-test. The causal relationships between factors were analyzed using multiple linear regression. The multicollinearity of the model was checked. Path analysis was performed to understand the BSC strategic maps based on the Palestinian HCWs' evaluations.

**Results:**

Out of 800 surveys, 454 (57%) were retrieved. No evaluation differences between physicians and nurses were found. The BSC-HCW1 model explains 22–35% of HCW loyalty attitudes, managerial trust, and perceived patient trust and respect. HCWs' workload time-life balance, quality and development initiatives, and managerial performance evaluation have a direct effect on improving HCWs' loyalty attitudes (β = 0.272, *P* < 0.001; β = 0.231, *P* < 0.001; β = 0.199, *P* < 0.001, respectively). HCWs' engagement, managerial performance evaluation, and loyalty attitudes have a direct effect on enhancing HCWs' respect toward managers (β = 0.260, *P* < 0.001; β = 0.191, *P* = 0.001; β = 0.135, *P* = 0.010, respectively). Quality and development initiatives, HCWs' loyalty attitudes, and workload time-life balance had a direct effect on improving perceived patient respect toward HCWs (β = 254, *P* < 0.001; β = 0.137, *P* = 0.006, β = 0.137, *P* = 0.006, respectively).

**Conclusion:**

This research shows that it is important to improve low-performing indicators, such as the duration of time HCWs spend with patients, their knowledge of medications and diseases, the quality of hospital equipment and maintenance, and the inclusion of strengths and weaknesses in HCWs' evaluations, so that HCWs are more loyal and less likely to want to leave. For Palestinian hospital managers to be respected more, they must include HCWs in their action plans and explain their evaluation criteria. Patients will respect Palestinian HCWs more if they prioritize their education and work quality, spend more time with patients, and reflect more loyalty. The results can be generalized since it encompassed 30% of Palestinian hospitals from all categories.

## 1. Introduction

The Occupied Palestinian Territories (OPT) health care system is regarded as unstable and incoherent (1). This refers to the current political and economic obstacles that prevent the progress of the Palestinian health care industry (2). In addition, the administrative hospitals in the OPT come in a wide range of various types. There are 28 public hospitals, 39 hospitals operated by non-governmental organizations (NGOs), 17 private hospitals, two military hospitals, and one hospital operated by the United Nations Relief and Works Agency for Palestine Refugees in the Near East (UNRWA) (3). Geographically, there are 50 hospitals in the West Bank, seven in eastern Jerusalem, and 30 in the Gaza Strip (3). The proportion of beds by administrative type is ~59%: 26% are NGOs, 14% are private, and 1% are UNRWA, while military hospitals are not yet active (4).

### 1.1. Performance evaluation in hospitals

Due to the restricted capacity of hospital beds and the increased psychological stress of health care workers (HCWs) during the coronavirus (COVID-19) pandemic (5, 6), the COVID-19 pandemic incurred additional expenditures for the global health care system. In the era of COVID-19, there is still a dearth of data that would aid health care managers and policymakers in boosting the quality of future health care delivery and learning (7). Prior to the pandemic, it was crucial that the health care system use key performance indicators (KPIs) for a variety of reasons. First, evaluations of patient and HCW satisfaction were improved. Second, the application of KPIs increases efficiency, effectiveness, and financial performance while adapting to new technologies and ideas. Third, the use of KPIs improves productivity and profitability (8, 9). Keeping track of KPIs during a pandemic is especially important for health care organizations (HCOs), as it may assist in identifying areas that need urgent attention and reinforcement (10).

**Figure 1:**
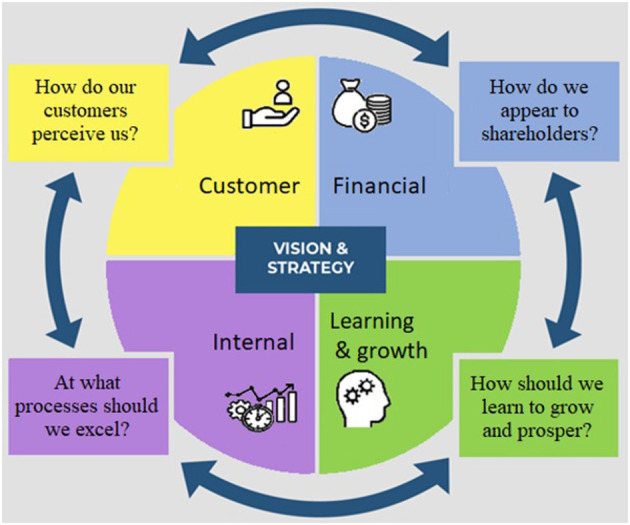
First generation BSC Perspectives [source: (13) with adaptation].

The balanced scorecard (BSC) is one of the strategic management tools that has been applied internationally by many hospitals and employed KPIs for the performance evaluation of HCOs (11). In their initial 1992 proposal for the BSC, Norton and Kaplan combined four perspectives: financial, customer, internal process, and knowledge and growth (12) (Figure 1). Other BSC implementations called it learning and development (11). The external perspective was then deemed the fifth BSC pillar, which includes sustainability and social aspects (14). In our BSC systematic review (15), we found that there is a need to add the managerial perspective in addition to the external perspective to the BSC design, which means having to balance the focus on six perspectives. Additionally, we concluded that there is a need to have a separate consideration when evaluating knowledge and technology subdimensions when considering the knowledge and growth perspective (16–18).

**Figure 2:**
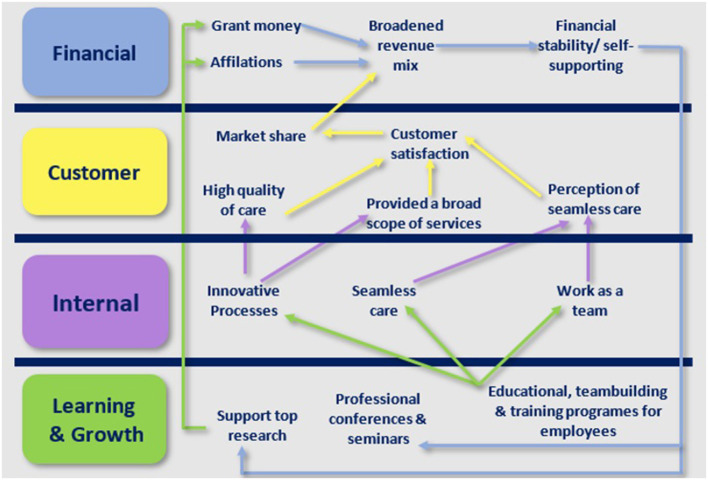
Strategic map of the Duke University health system [source: (20) with adaptation].

In the second generation of the BSC, researchers established causal relationships between the KPIs of these four perspectives (19) (see Figure 2). This network of causal models was referred to as the BSC strategic map. The third generation, which included goals and action plans for each KPI, was then developed. Most current PE models concentrate on the internal perspective and ignore other essential perspectives. Two characteristics differentiate the BSC from other management tools. As the first component, it enables managers to focus on both financial and non-financial aspects, thus providing a comprehensive approach to PE. Second, the BSC is more than a planning or PE instrument; it is also a strategic management instrument. It assigns KPIs connected to the HCO strategy (13, 19). Other PE systems, such as total quality management (TQM), are not as comprehensive (21).

**Figure 3:**
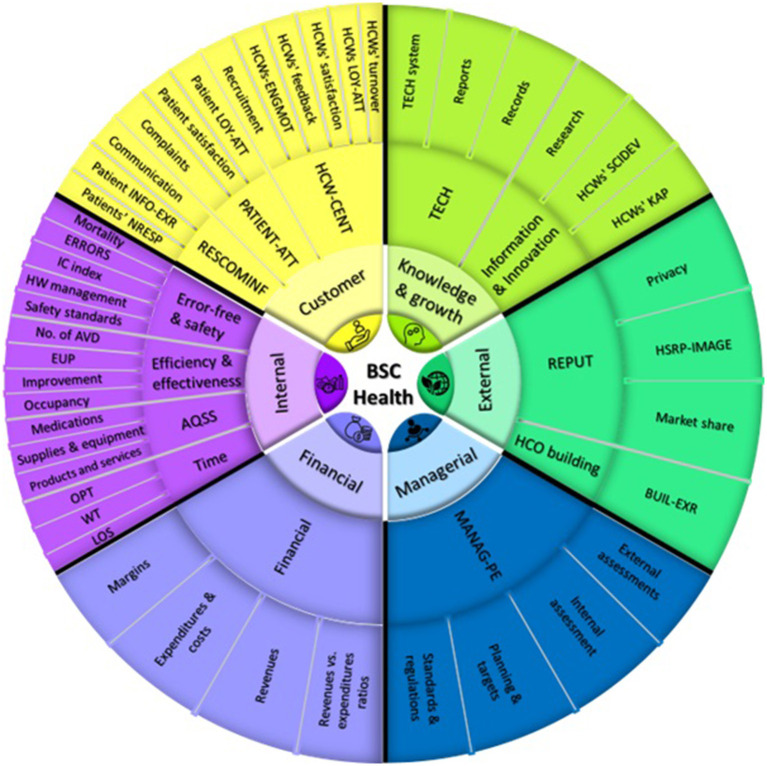
A summary of BSC perspectives in health care and their contents [source: own elaboration (11) with adaptation]. BSC, balanced scorecard; HCWs, health care workers; HCOs, health care organizations; IC, infection control; HW, health waste; WT, waiting time; LOS, length of stay; KAP, knowledge, attitudes, and practices; TECH, technology; HSRP-IMAGE, hospital social responsibility perceived image; ERRORS, errors, accidents, and complications; No. of AVD, number of admissions, visits, and diseases; EUP, efficiency, utilization, and productivity; AQSS, availability and quality of supplies and services; OPT, operation processing time; RESCOMINF, patient need response, communication, and information provision; PATIENT-ATT, patient attitude; HCW-ENGMOT, health care workers' engagement and motivation; HCW-CENT, health care workers' centrality; MANAG-PE, managerial tasks and performance evaluation; HCW-SCIDEV, health care workers' scientific development; INFO-EXR, information experience; LOY-ATT, loyalty attitudes; BUIL-EXR, building experience; REPUT, community and reputation; NRSP, needs response.

Additionally, our systematic review of the BSC showed that HCOs' financial performance improved when the BSC was put into place (22). We also found that BSC was helpful in improving the rate of patient satisfaction. However, BSC had a minor effect on the satisfaction rate of HCWs (15). On the other hand, we found heterogeneity in the KPIs, dimensions, and perspectives used in BSC implementations, as well as how they were categorized into groups. To resolve this issue, we conducted a second BSC systematic review (11) in which 797 KPIs from 36 BSC implementations were extracted, classified, and regrouped. This resulted in Figure 3. The dimensions and KPIs that emerged from Figure 3 in tandem with examining 77 causal linkages in 34 studies in the literature (11, 14, 16, 22–52) served as the basis for developing an instrument that we designed to specifically engage HCWs in BSC implementations (BSC-HCW1) (18).

### 1.2. HCWs' engagement

Regular participation by HCWs in determining how their work is performed (53), involvement in improvement suggestions (53), goal setting (53), planning (53), performance monitoring (53), leadership engagement (54), quality improvement projects (55), and research are just a few of the numerous types of HCW participation (55). Globally, the involvement of physicians and nurses in healthcare is considered an essential strategy since they are mostly known as frontline health care personnel (55–57). The engagement of HCWs results in enhancements to HCWs' wellbeing (55), levels of perceived patient care quality (55), patient outcomes (56), data quality (53), efficiency (53), innovation (53), HCWs satisfaction (53, 55, 58), patient satisfaction (53), performance (53), and decreased levels of unscheduled time off work (55). However, research indicates that the nursing voice is often overlooked (57). A review concluded that physicians' involvement techniques include senior leadership support and data-driven quality improvement (28). Additional strategies included the allocation of time, resources, and training for quality improvement work; incentives; the clarification of organizational goals; and the development of promotion pathways (28). Furthermore, HCWs' engagement during the pandemic was even considered more vital for HCOs (59, 60).

In addition to the importance of engaging HCWs in enhancing the performance of HCOs worldwide, in OPTs specifically, the health care system's exploited challenges have emphasized the significance of a deeper knowledge of the Palestinian HCW perspective. A BSC implementation (61) determined that there are few validated instruments to assess management practices in low- and middle-income countries (LMICs), and none of these instruments are related to the BSC. In OPTs, there is also insufficient research on PE for hospitals. As a result, in a previous study, we validated the first instrument designed to engage HCWs in a comprehensive evaluation of BSC perspectives and dimensions (BSC-HCW1). The evaluation of financial, customer, internal process, knowledge and development, and managerial perspectives and dimensions, based on aspects that are directly pertinent to HCWs' needs. In this study, we aim to (1) engage HCWs in evaluating Palestinian hospitals based on BSC perspectives and dimensions, (2) compare the differences between physicians' and nurses' evaluations of BSC dimensions at Palestinian hospitals, and (3) determine which experiences predict HCW attitudes and which HCWs experience impact each other. These aims will allow us to draw recommendations for policy makers to improve the PE of hospitals in OPTs.

### 1.3. Theoretical framework

**Figure 4:**
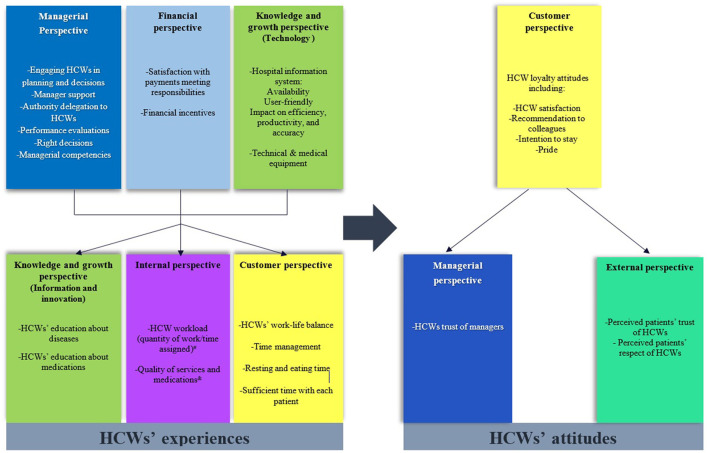
The theoretical framework for the impact of HCWs' experiences on their attitudes based on BSC perspectives (source: own elaboration). ^#^Workload loaded with work-life balance in customer perspective items in the workload time-life balance (WTLB) factor; ^&^Loaded with knowledge and growth items in the quality and development initiatives (QUALDEV) factor.

Figure 4 is the theoretical framework that represents the BSC strategic map from the HCWs' point of view. We hypothesize that managerial experience has an important role in improving HCWs' experiences related to knowledge and education, workload, time management, time spent with each patient, life balance, and quality of services and medications. Additionally, managerial experience plays an important role in improving HCWs' attitudes, such as their satisfaction, loyalty, and pride attitudes, collectively called loyalty attitudes. Additionally, managerial experience influences the external perspective, including patients' perceived trust and respect of HCWs. Finally, we believe that managerial experience also influences HCWs' trust in their managers.

In parallel, we hypothesize that HCWs' beliefs regarding their payments suiting their responsibilities and the incentives they receive, as well as HCWs' experiences regarding hospital information systems and hospital equipment, influence the above-mentioned HCWs' attitudes. On the other hand, we hypothesize that improving HCWs' education and knowledge about medications and diseases, the quantity of assigned work, the quality of services and medications, their ability to achieve work-life balance, time management, time spent with each patient, and resting and eating time will improve all HCWs' attitudes, including their satisfaction, recommendation, intention to stay, pride, managerial trust, and perceived patient trust and respect. Finally, we believe that improving HCWs' loyalty attitudes will improve their trust in their managers and the perceived respect and trust of patients.

## 2. Methodology

### 2.1. Study design and sample

This cross-sectional design is a subset of a broad multisite project (11, 15–18). The project's overall aim is to use BSC perspectives and dimensions to include a wide range of stakeholders in the strategic improvement of Palestinian hospitals' performance. This article focuses on engaging Palestinian health care workers, particularly physicians and nurses. The reporting of this study follows the Strengthening the Reporting of Observational Studies in Epidemiology (STROBE) criteria (62).

### 2.2. Sample calculation

Due to geographical and logistical limitations, we were only able to choose 18 hospitals in the OPT for this research. However, we took into consideration the fact that our hospital sample consists of hospitals with various sizes, locations, and administrative styles. Maximum variation sampling was used for this objective (3). The number of hospitals and beds in each administrative category and governorate were considered while selecting a sample of hospitals. Patient samples were also picked easily. HCWs in the selected departments were approached during the visit and asked if they would be interested in participating in the study. The sample size was determined using the Steven K. Thompson sample size equation (63),


(1) $$\begin{array}{l} n=\frac{N\;\times p\left(1-p\right)}{\left[N\;-1\;\times \left(d^{2\;}÷z^{2}\right)\right]+p\left(1\;-p\right)} \end{array}$$

where *n* is the sample size, *N* is the population size, *p* is the estimated population variability (0.5), *d* is the margin of error (0.05), and the *z*-score is at the 95% confidence interval (1.96). Research indicates that 36,809 HCWs are employed in Palestinian hospitals (64). Hence, the minimum sample size needed was found to be 381 HCWs. The authors were worried about the low response rate as a result of the pandemic's effect on hospitals and the HCWs' heavy workload, a perception shared by other studies (65, 66). In addition, there is a poor response rate of physicians relative to the rest of the population (67, 68). Therefore, 800 questionnaires were delivered as a result.

### 2.3. Measures

We employed the validated survey BSC-HCW1. The Arabic version was utilized. The validation of the BSC-HCW1 at Palestinian hospitals resulted in 28 items and nine factors. The six experience factors are the management performance evaluation (MANAG-PE), financial incentives (FIN), quality and development initiatives (QUALDEV), technology (TECH) factor, HCWs' engagement (ENG), and workload time-life balance (WTLB). The three HCWs' attitudes are the HCWs' loyalty attitudes (LOY-ATT), the perceived patient respect and trust of HCWs, and the trust of HCWs toward their direct managers (MTR).

### 2.4. Data collection

The first author and four medical students from An-Najah National University were responsible for the data collection. Before beginning data collection, the main author led a training session for the medical students that lasted for 3 h and covered a brief about BSC, guidelines for data collection, and ethical considerations. The team members were assigned duties and hospitals depending on where they resided: eastern Jerusalem, northern, middle, and southern West Bank. The Gaza Strip was omitted for political and pragmatic inaccessibility reasons. In addition, five institutions were omitted from the list: two military hospitals that had not yet opened, one mental hospital, and two rehabilitation hospitals.

To prevent non-response bias, between January and October 2021, printed surveys were given to respondents instead of emailing the questionnaires (69). To avoid response bias (69), the “I do not know (neutral)” option was introduced, given that experiences and attitudes might sometimes be ambiguous (70). Second, to guarantee that the number of missing responses had been reduced to a minimum, the data collectors reviewed the retrieved surveys. In the case of missing items, they drew the participant's attention to record a response. If any missing responses were discovered during data input, they were recorded as I do not know. The inclusion and exclusion criteria were a Palestinian doctor or nurse of either gender who had worked at any of the evaluated hospitals for at least 3 months. Emergency medicine, internal medicine, surgery, gynecology, and pediatrics were among the departments covered.

### 2.5. Statistical analysis

The first author coded the data, and then the normality of the data was examined using the Shapiro–Wilk test. In addition, frequency calculations were carried out for the categorical patient sociodemographic items. On the 3-point Likert scale, “No” responses were scored as 0, “Yes” responses as 100, and “I do not know” responses as 50. Each question's frequency was determined. Then, the mean score and standard deviation (SD) of each factor in both the physician and nurse categories were determined by calculating the average score for the underlying questions (48). After piloting, Cronbach's alphas for the scale, subscales, and factors were computed.

We used the Mann–Whitney *U*-test to test the differences between the physicians' and nurses' evaluations of the BSC-HCW1 factors. The strength of the relationship between the independent variables or between the dependent and independent factors was examined using Pearson's correlation (*r*). Then, *r* was defined as negligible when *r* < 0.2, low when *r* = 0.2–0.49, moderate when *r* = 0.5–0.69, high when *r* = 0.7–0.85, and very high when *r* = 0.86–1.00 (71).

Multiple linear regression was used to examine the causation link of the independent variable factors on each dependent variable factor, with a *P*-value <0.05 for statistical significance and 95% confidence interval (CI). The residual plots were examined for normal distribution and linearity. The Durbin-Watson (DW) test was calculated, then the lower and upper critical values (DL and DU) were checked to examine autocorrelation, also known as serial correlation (72). At 1% level of significance, DL = 1.61 and DU = 1.74. The acceptance range (DU, 4-DU) = (1.74, 2.26). In addition, we investigated the model's multicollinearity. Multicollinearity occurs when independent variables in a regression model are correlated, which is problematic since independent variables should be unrelated. Multicollinearity was identified if any of the threshold values shown below (73, 74) were exceeded: 1- the Pearson correlation between variables was >0.70, 2- a variance inflation factor (VIF) >10, 3- a condition index >30, and 4- a variance decomposition proportion (VDP) for two or more predictors that was more than 0.80.

Finally, path analysis is considered a method for enhancing conceptual comprehension and illustration of regression findings, particularly in complicated models (64). Therefore, to develop the strategic map of BSC factors from the HCWs' perspective, we conducted a path analysis for the dependent and independent variables of BSC-HCW1 collectively. To arrive at the best fit model, we kept the regressions that were significant, utilized the modification indices, and used the most used fit indices of the competing models; a minimum discrepancy divided by its degrees of freedom (χ^2^/df) <5 and closer to zero, a *P*-value more than 0.05, the goodness-of-fit index (GFI), the comparative fit index (CFI), the Tucker–Lewis index (TLI), and cutoff values of ~0.95. Additionally, a root mean square error of approximation (RMSEA) value <0.06 and a standardized root mean square residual (SRMR) value <0.08 were sought (75, 76). Based on the final resulting best fit model, we assessed the standardized direct and indirect impacts of factors on each other. Statistical Package for the Social Sciences (SPSS) version 21.0 was used for all the tests except the path analysis, which we performed with IBM Amos Graphics version 23.0. Additionally, R version (3.1.0) was used to create the correlogram.

### 2.6. Ethical considerations

The Research and Ethics Committee of An-Najah National University's Faculty of Medicine and Health Sciences issued the Institutional Review Board (IRB) with a reference code number on May 31, 2020 (Mas, May/20/16). After that, we obtained permission from the Palestinian Ministry of Health to perform the study at public hospitals. The request was then sent to each hospital separately. Requests were made to 15 West Bank hospitals and three Jerusalem hospitals between June and December 2020. In accordance with the ethical standards outlined in the Declaration of Helsinki, all of the HCWs gave written, informed permission to participate in the research (77). The confidentiality and anonymity of the data were guaranteed to the HCWs. Participation in the research was optional, and all HCWs were made aware of this fact and given the opportunity to withdraw at any time.

## 3. Results

As the study was conducted during the COVID-19 pandemic, obtaining hospital permissions took 9 months. Only 15 of the 18 hospitals agreed to participate in the study. The information was gathered between January and October of 2021. The hospital that was included in the pretest was excluded. Then, we distributed 800 surveys to the remaining 14 hospitals, from which we collected 454 valid questionnaires (response rate of 57%), which is higher than the required sample size of 381. The data did not follow a normal distribution. In the subsequent phases, non-parametric tests, notably Spearman correlations and Mann–Whitney *U*-tests, were used.

### 3.1. Participant characteristics

The characteristics and sociodemographic characteristics of the HCWs are shown in Table 1.

**Table 1:** Sociodemographic characteristics of HCWs (N = 454).

**Characteristics**	***N***	**%**
**Age**		
20–29 years	198	43.6
30–39 years	163	35.9
40–49 years	59	13
50–59 years	26	5.7
60 years or above	8	1.8
**Gender**		
Female	232	51.1
Male	222	48.9
**Department**		
Mixed	18	4.0
Pediatric	73	16.1
Internal medicine	81	17.8
Surgery	98	21.6
Emergency	91	20.0
Gynecology	93	20.5
**Years of experience**		
0–2 years	149	32.8
3–5 years	107	23.6
6–9 years	79	17.4
10–13 years	45	9.9
More than 14 years	74	16.3
**Income**		
2,000–3,000 NIS	108	23.8
3,001–4,000 NIS	129	28.4
4,001–5,000 NIS	101	22.2
5,001–6,000 NIS	50	11.0
Higher than 6,000 NIS	66	14.5
**Profession**		
Doctor	156	34.4
Nurse	298	65.6

NIS, New Israeli Shekel; UNRWA, The United Nations Relief and Works Agency for Palestine Refugees in the Near East; NGO, non-governmental organization; mixed, only in one hospital were the nurses not specified to work in one department and were rotated between different departments.

### 3.2. Descriptive analysis

Table 2 displays the proportion of responses per question, as well as the means and standard deviations of the factors. The MTR factor had the greatest mean score (87.4 ± 24.7), while the FIN factor had the lowest mean score (52.4 ± 25.2). Cronbach's alpha for all factors was acceptable. Cronbach's alpha for the BSC-HCW1 was 0.898, for the experience subscale was 0.872, and for the attitude subscale was 0.761.

**Table 2:** Descriptive statistics of factors and underlying questions (N = 454).

				**Cronbach's Alpha**		**Descriptive statistics**		
	**Factor**	**Q**	**Question**	**Factor**	**Subscale**	**No (%)**	**Yes**^!^ **(%)**	**Mean (**±**SD)**
IV	FIN	Q1	I receive financial incentives based on my performance	0.671	0.872	75.1	20.5	52.4 (±25.2)
		Q2	I feel that my salary suits my responsibilities and competencies			63.4	32.4	
		Q3	I believe that hospital information system interface is user friendly	0.837		28.4	62.8	80.6 (±23.7)
	TECH	Q4	I believe that hospital information system and technology at this hospital makes generating reports easier, faster, and more accurate			24.9	68.1	
		Q5	This hospital has a technology/information system			23.3	72.5	
		Q6	I believe that hospital information system and technology at this hospital makes my work efficient and productive			24.9	65.4	
		Q7	The quantity of work assigned to me is reasonable with the time given	0.743		37.4	59	74 (±23.5)
	WTLB	Q8	I have sufficient time to rest and eat during my working day			26.9	70.3	
		Q9	I am able to make a work-life balance and a good time management			31.7	62.8	
		Q10	I am able to spend a sufficient time with each patient			52.6	44.5	
	QUALDEV	Q11	The hospital provides me education on medication updates that is related to my specialty	0.801		35.7	56.6	75.9 (±22.8)
		Q12	The hospital provides me education updates regarding the diseases in my specialty			39.9	55.5	
		Q13	The hospital medications and disposables are of high quality			25.1	66.5	
		Q14	The hospital equipment helps me in offering high quality medical services for patients			37.9	56.4	
		Q15	Quality is top priority at this hospital			24.7	66.1	
	HCW-ENG	Q16	My manager engages me in the planning and taking decision process	0.703		38.1	54	76.6 (±23.8)
		Q17	I am given enough authority and power to make decisions in my position			31.7	59	
		Q18	My manager understands and adequately support me when I face an urgent hard situation			22.5	68.7	
	MANAG-PE	Q19	My direct superiors explain and discuss the strengths and weaknesses in my assessment with me	0.783		35.2	57.7	77.5 (±22.6)
		Q20	I believe that my superiors are taking the right decisions in work which supports the hospital strategy			20	73.1	
		Q21	I believe that my superiors have the required competencies for their positions			25.1	66.1	
		Q22	I believe that my assessment is fair and reflects my actual performance compared to your colleagues			29.5	56.8	
DV	MTR	Q23	I trust what my direct manager tells or promises me with	-	0.761	15.6	77.8	87.4 (±24.7)
	PTR	Q24	I belief that patients respect healthcare workers at this hospital and trust them	-		27.8	62.6	78.3 (±29.5)
	LOY-ATT	Q25	I believe and feel that I want to keep working in this hospital for several years	0.774		27.3	61.7	80.9 (±21.4)
		Q26	I recommend this hospital to other colleagues or praise the hospital			22	67	
		Q27	I believe and feel that my overall satisfaction is high			32.2	57.5	
		Q28	I am proud to work with this hospital			12.1	78	

MANAG-PE, management performance evaluation; HCW-ENG, health care workers' engagement; FIN, financial incentives; QUALDEV, quality and development; TECH, technology; WTLB, workload time-life balance; MTR, HCWs trusting their managers; PTR, perceived patient respect and trust attitudes toward health care workers; LOY-ATT, loyalty attitudes.

-, single item-factor.

^!^The rest answered I do not know.

### 3.3. Variance analysis for physicians' and nurses' evaluations

The variance analysis showed that the mean ranks for the nurses' evaluations were higher than those for physicians except for two factors: the FIN and WTLB. However, none of these differences were significant (see Table 3).

**Table 3:** Variance analysis between physicians' and nurses' evaluations for BSC-HCW1 factors.

**Factors**	**Mean rank**		***Z*-score**	***P*-value**
**Nurses (*****N*** = **298)**	**Physicians (*****N*** = **156)**			
MANAG-PE	233.22	216.56	−1.319	0.187
HCW-ENG	228.81	225.01	−0.304	0.761
FIN	223.17	235.77	−1.092	0.275
QUALDEV	235.23	212.74	−1.77	0.077
TECH	230.45	221.87	−0.698	0.485
WTLB	220.41	241.04	−1.626	0.104
MTR	229.08	224.49	−0.488	0.625
PTR	228.84	224.94	−0.516	0.606
LOY-ATT	229.71	223.28	−0.351	0.726

MANAG-PE, management performance evaluation; HCW-ENG, health care workers' engagement; FIN, financial incentives; QUALDEV, quality and development; TECH, technology; WTLB, workload time-life balance; MTR, HCWs trusting their managers; PTR, perceived patient respect and trust attitudes toward health care workers; LOY-ATT, loyalty attitudes.

### 3.4. Correlations

**Figure 5:**
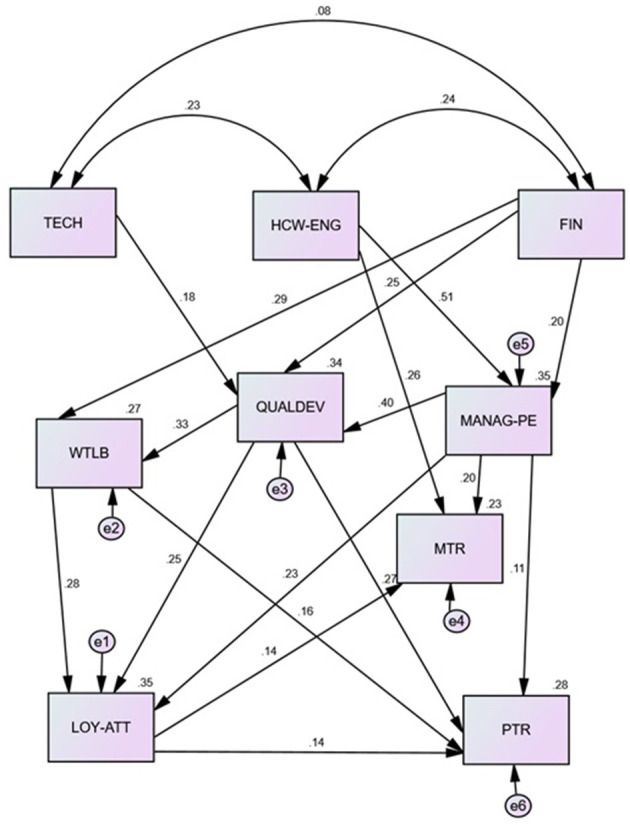
Spearman correlation (*r*) between BSC-HCW1 factors (source: own elaboration). *r* < 0.2, negligible; (*r* = 0.2–0.49), low; (*r* = 0.5–0.69), moderate; (*r* = 0.7–0.85), high; (*r* = 0.86–1.00), very high. MANAG-PE, management performance evaluation; HCW-ENG, health care workers' engagement; FIN, financial incentives; QUALDEV, quality and development; TECH, technology; WTLB, workload time-life balance; MTR, HCWs trusting their managers; PTR, perceived patient respect and trust attitudes toward health care workers; LOY-ATT, loyalty attitudes.

Pearson correlations between the factors were either negligible or weak, except between MANAG-PE and two factors; HCW-ENG and QUALDEV were moderate. See the correlogram in Figure 5. None of the correlations were high or very high, which reflects the distinction and the discriminant validity of the scale factors (71).

### 3.5. The causal model

#### 3.5.1. Impact of HCWs' experiences on loyalty attitudes

The plot of residuals in SPSS revealed a normal distribution and linearity. The DW was 1.807 which lies in the accepted range. Multiple linear regression results showed that 35.6% of the variance in HCWs' LOY-ATT can be collectively predicted by six experience factor types [*F*_(6.447)_ = 42.825, *P* < 0.001]. Looking at the unique individual contributions of the predictors, the results show that WTLB (β = 0.272, *P* < 0.001), QUALDEV (β = 0.231, *P* < 0.001), and MANAG-PE (β = 0.199, *P* < 0.001) positively predicted LOY. However, the FIN, HCW-ENG, and TECH effects were not significant (see Table 4). This model is free of multicollinearity since all correlations were <0.7, and the VIF range was 1.153–1.829, the highest condition index was 15.220, and no (VDP) for two or more predictors was more than 0.80.

**Table 4:** Evaluation of the causal effect of HCWs' experiences on their attitudes.

**Factor**	**Standardized coefficients**	***T***	***P*-value**	**CI**
β			
**Factors affecting LOY-ATT**		
MANAG-PE	0.199	3.906	*P* < 0.001	[0.094, 0.283]
HCW-ENG	0.030	0.662	0.509	[−0.054, 0.109]
FIN	0.048	1.108	0.269	[−0.031, 0.113]
QUALDEV	0.231	4.707	*P* < 0.001	[0.126, 0.307]
TECH	0.039	0.951	0.342	[0.037, 0.107]
WTLB	0.272	6.081	*P* < 0.001	[0.167, 0.327]
Model summary	*R*^2^ adjusted = 0.356, *F*_(6.447)_ = 42.825, *P* < 0.001			
**Factors affecting MTR**				
MANAG-PE	0.191	3.351	0.001	[0.086, 0.331]
HCW-ENG	0.260	5.148	*P* < 0.001	[0.167, 0.373]
FIN	0.019	0.395	0.693	[−0.073, 0.110]
QUALDEV	0.067	1.208	0.228	[−0.045, 0.189]
TECH	−0.055	−1.246	0.213	[−0.149, 0.033]
WTLB	−0.049	−0.956	0.339	[−0.157, 0.054]
LOY-ATT	0.135	2.601	0.010	[0.038, 0.274]
Model summary	*R*^2^ adjusted = 0.224, *F*_(7.446)_ = 19.668, *P* < 0.001			
**Factors affecting PTR**				
MANAG-PE	0.106	1.937	0.053	[−0.002, 0.279]
HCW-ENG	−0.016	−0.337	0.737	[−0.139, 0.098]
FIN	0.090	1.962	0.050	[0.000, 0.210]
QUALDEV	0.254	4.803	*P* < 0.001	[0.194, 0.463]
TECH	0.021	0.496	0.620	[−0.078, 0.131]
WTLB	0.135	2.755	0.006	[−0.049, 0.290]
LOY-ATT	0.137	2.751	0.006	[0.054, 0.324]
Model summary	*R*^2^ adjusted = 0.284, *F*_(6.446)_ = 26.726, *P* < 0.001			

MANAG-PE, management performance evaluation; HCW-ENG, health care workers' engagement; FIN, financial incentives; QUALDEV, quality and development; TECH, technology; WTLB, workload time-life balance; MTR, HCWs trusting their managers; PTR, perceived patient respect and trust attitudes toward health care workers; LOY-ATT, loyalty attitudes.

#### 3.5.2. Impact of HCWs' experiences on trusting management attitudes

The plot of residuals in SPSS revealed a normal distribution and linearity. The DW value was 1.859 which lies in the accepted range. Multiple linear regression results showed that 22.4% of the variance in HCWs' LOY-ATT can be collectively predicted by six experience factor types [*F*_(7.446)_ = 19.668, *P* < 0.001]. Looking at the unique individual contributions of the predictors, the results show that HCW-ENG (β = 0.260, *P* < 0.001), MANAG-PE (β = 0.191, *P* = 0.001), and LOY-ATT (β = 0.135, *P* = 0.010) positively predicted MTR. However, the FIN, QUALDEV, WTLB, and TECH effects on the MTR were not significant (see Table 4). This model is free of multicollinearity since all correlations were <0.7, and the VIF range was 1.155–1.891, the highest condition index was 16.951, and no (VDP) for two or more predictors was more than 0.80.

#### 3.5.3. Impact of HCWs' experiences on perceived patients' respect and trust attitude

The plot of residuals in SPSS revealed a normal distribution and linearity. The DW value was 1.859 which lies in the accepted range. Multiple linear regression results showed that 28.4% of the variance in HCWs' LOY-ATT can be collectively predicted by six experience factor types [*F*_(6.446)_ = 26.726, *P* < 0.001]. Looking at the unique individual contributions of the predictors, the results show that QUALDEV (β = 0.254, *P* < 0.001), WTLB (β = 0.135, *P* = 0.006), and LOY-ATT (β = 0.137, *P* = 0.006) positively predicted PTR. However, the MANAG-PE, HCW-ENG, FIN, QUALDEV, and TECH effects on PTR were not significant (see Table 4). This model is free of multicollinearity since all correlations were <0.7, and the VIF range was 1.155–1.891, the highest condition index was 16.951, and no (VDP) for two or more predictors was more than 0.80.

#### 3.5.4. Path analysis

**Figure 6:**
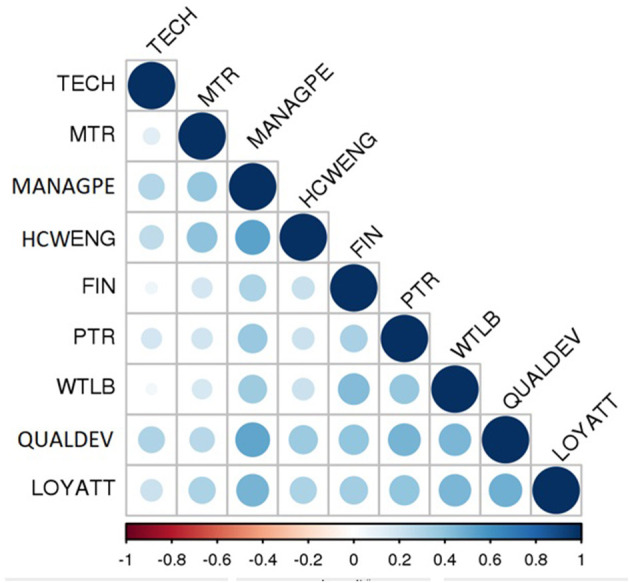
The resulting strategic map of Palestinian HCWs based on the path analysis of BSC-HCW1 factors (source: own elaboration). The numbers on the straight lines reflect the standardized regression weights (total effect). The numbers on the curved lines represent the correlations between experience factors. MANAG-PE, management performance evaluation; HCW-ENG, health care workers' engagement; FIN, financial incentives; QUALDEV, quality and development; TECH, technology; WTLB, workload time-life balance; MTR, HCWs trusting their managers; PTR, perceived patient respect and trust attitudes toward health care workers; LOY-ATT, loyalty attitudes.

The goodness of fit indices for the best resulting model are shown in Figure 6. All of them met the conditions of a good fit model, except for the *P*-value. Additionally, all regressions illustrated between factors in this final model were significant. In general, this strategic map model predicts 35.2% MANAG-PE, 34.3% QUALDEV, and 26.8% WTLB. Regarding the prediction of attitudes, this model predicts 35.1% of LOY, 28.2% of PTR, and 22.7% of MTR. On the other hand, the path analysis enabled us to understand the direct and indirect effects of the BSC-HCW1 factor, including experiences and attitudes on each other (see Table 5).

**Table 5:** Direct and indirect effects of BSC-HCW1 factors on each other based on path analysis of the best final resulting model.

**Factor**	**FIN**		**TECH**		**HCW-ENG**		**MANAG-PE**		**QUALDEV**		**WTLB**		**LOY-ATT**	
MANAG-PE	**0.202**				**0.511**								
QUALDEV	**0.247**	*0.081*	**0.177**			*0.205*	**0.401**						
WTLB	**0.294**	*0.107*		*0.058*		*0.067*		*0.205*	**0.326**				
LOY-ATT		*0.242*		*0.061*		*0.188*	**0.229**	*0.067*	**0.253**	*0.092*	**0.282**		
PTR		*0.210*		*0.066*		*0.150*	**0.112**	*0.188*	**0.273**	*0.101*	**0.158**	*0.040*	**0.142**
MTR		*0.073*		*0.008*	**0.262**	*0.127*	**0.199**	*0.150*		*0.047*		*0.038*	**0.137**
Model summary (SMC)	MANAG-PE = 0.352, QUALDEV = 0.343, WTLB = 0.268, LOY-ATT = 0.351, PTR = 0.282, MTR = 0.227													
Goodness of fit indices	χ^2^/df = 2.371. *P*-value = 0.002. GFI = 0.983. CFI = 0.979. TLI = 0.954. RMSEA = 0.055. SRMR = 0.0348.													

Bold, standardized direct effect; Italic, standardized indirect effect; empty cells, neither direct nor indirect effect.

MANAG-PE, management performance evaluation; HCW-ENG, health care workers' engagement; FIN, financial incentives; QUALDEV, quality and development; TECH, technology; WTLB, workload time-life balance; LOY-ATT, loyalty attitudes; MTR, HCWs trusting their managers; PTR, perceived patient respect and trust attitudes toward health care workers; SMC, squared multiple correlations; χ^2^/df, minimum discrepancy divided by its degrees of freedom; GFI, goodness-of-fit index; CFI, comparative fit index; TLI, Tucker–Lewis's index; RMSEA, root mean square error of approximation; SRMR, standardized root mean square residual.

First, the TECH effect on HCWs' attitudes in general was neglected but had a direct impact on QUALDEV. Second, HCW-ENG and FIN had a direct impact on MANAG-PE. Additionally, HCW-ENG had direct and indirect effects on MTR and only indirect effects on LOY-ATT and PTR, which reflects that MTR and MANAG-PE work as mediators. HCW-ENG also had a direct effect on MANAG-PE. Fourth, MANAG-PE had a direct impact on QUALDEV and an indirect effect on MTR and PTR, which reflects that QUALDEV acts as a mediator. Fifth, QUALDEV had a direct impact on WTLB. Although the FIN factor did not have a significant direct effect on HCWs' attitudes, the path analysis revealed that FIN had an indirect effect on LOY-ATT and PTR, which reflects that MANAG-PE, WTLB and QUALDEV act as mediators for the effect of FIN on LOY-ATT and PTR.

## 4. Discussion

### 4.1. Discussion of the main results

This study was successful in achieving its three aims. First, we engaged HCWs in evaluating Palestinian hospitals based on the BSC perspectives and dimensions. The results revealed that there are low-performing factors and KPIs that require better consideration from Palestinian hospital managers to improve. Specifically, financial incentives, sufficient time spent with patients, HCWs' education updates on medications and diseases, hospital equipment quality and maintenance, the inclusion of strengths and weaknesses explanations in HCWs' appraisals, HCWs' intent to stay or leave, and their satisfaction rate. As per our second aim, we compared the differences between physicians' and nurses' evaluations of BSC dimensions at Palestinian hospitals. We found no differences among these two categories regarding their evaluations of experiences and attitudes. Third, we assessed which HCWs' experiences predicted their attitudes and which experiences influenced each other. In summary, the most significant factors that affect HCWs' loyalty attitudes and need for better improvement in Palestinian hospitals were workload time-life balance, including the time spent with patients; quality and development initiatives, including equipment quality and maintenance; and managerial performance evaluations, including HCWs' appraisal clarifications. To enhance the respect of HCWs toward hospital managers, managers should enhance HCWs' engagement culture and HCWs' appraisal clarification. To improve the perceived respect by Palestinian patients toward HCWs, managers and HCWs have to focus on HCWs' education and quality improvements, followed by improving time spent with patients and HCWs' loyalty attitudes. The final best model of BSC-HCW1 showed a high fit adequacy for all indices except the *P*-value, which can be referred to as its sensitivity to data normality. The BSC-HCW1 model explains 22–35% of HCW loyalty, managerial trust, and perceived patient trust and respect. Neither multicollinearity nor autocorrelation were detected.

### 4.2. Comparison with studies

In comparison with other BSC implementations, reviews (11) revealed that most of the previous implementations did not consider engaging HCWs in the BSC implementations. The main focus was only on assessing the HCW satisfaction perspective without focusing on the other BSC perspectives. This finding reflects the significance of BSC-HCW1 utilization and the uniqueness of this investigation. On the other hand, we compared our results with those of other studies that evaluated BSC perspectives and dimensions as separate outcome measures.

#### 4.2.1. Managerial perspective

Our findings regarding the impact of managerial performance are compatible with a study (78) that found that competency-based management can promote nurses' enthusiasm, improve their satisfaction, reduce burnout, and improve patient satisfaction. On the other hand, a study revealed that better managerial engagement of physicians was also linked with higher physician satisfaction (52). Another study (28) found that most of the variance in HCWs' intention to stay attitude referred to managers respecting HCWs' opinions and engaging them in decision making. The results of these two studies are different from our results, which revealed that engagement itself did not have a direct impact on HCWs' loyalty attitudes but had a direct impact on HCWs' attitudes toward their direct managers and trusting them. In the same vein, a study revealed that HCW engagement enhanced the levels of perceived patient care quality (55), which matches our results that HCW engagement is a predictor for enhancing quality and development initiatives as well as improving the perceived respect and trust of patients toward HCWs.

#### 4.2.2. Financial perspective

The same is true regarding the impact of financial payments and motivations; our results are different from many reviews (22, 28, 29), which revealed that satisfaction with payment contributed to the greatest variance in HCW satisfaction. In our findings, the financial factor did not have a direct impact on HCWs' loyalty attitudes but had an indirect effect. This is because managerial evaluation, quality improvement and development, and workload time-life balance factors work as mediators.

#### 4.2.3. Knowledge and growth perspective

A study found that on-the-job training motivated 99.0% of HCWs (31). This result could be compatible with our findings that quality and development initiatives such as education programs on diseases and medications are predictors of HCWs' loyalty attitudes and the highest predictor of patient respect and trust. Regarding the technology perspective, the effect of technical and medical equipment on HCW satisfaction was found to improve the motivation of HCWs (26). However, in our analysis, technology did not have a direct impact on HCWs' attitudes. However, it had a direct impact on improving the quality and development factor.

#### 4.2.4. External perspective

Although social factors such as the community and patients' appreciation were frequently assessed, they were evaluated from the patients' point of view (30). We could not find studies measuring how this factor is perceived from the HCWs' side. Our study is one of the few studies that found that initiatives to improve quality and development, followed by HCWs' loyalty attitudes and workload time-life balance, were direct predictors of patient respect and trust. HCWs' financial incentives, HCW engagement, and managerial evaluation factors had an indirect effect on affecting patients' respect and trust.

#### 4.2.5. Internal perspective

A high workload and HCW shortage were found to negatively influence HCWs' satisfaction (26, 30, 32, 52). Workload time-life balance was found to positively affect HCWs' satisfaction (36). This is similar to our findings that this workload time-life balance expected the greatest variance in HCWs' loyalty attitudes. Additionally, it had a role in predicting patient respect and trust.

#### 4.2.6. Customer perspective

However, BSC implementations focused on the assessment of HCW satisfaction. Other HCWs' loyalty attitudes were rarely considered in such evaluations (11). Additionally, the experience factors affecting these attitudes were also not studied (11). Our study agrees with a study highlighting that a satisfaction survey should include key contextual factors affecting it (39). However, our study is different from other studies that consider HCW satisfaction as a separate outcome measure predicting other loyalty attitudes (42). Intent to stay or leave was also evaluated in studies as a separate outcome measure, specifically when turnover cannot be measured directly (43, 52). A study (45) revealed a negative relationship between job satisfaction and nurses' intention to quit their current hospital. On the other hand, pride attitude was a predictor of healthy working conditions (47). In our survey, HCWs' satisfaction, intent to stay or leave, recommendations to colleagues, and feelings of pride were considered loyalty attitude factors, which are directly affected by managerial performance, quality and development initiatives, and HCWs' workload time-life balance and indirectly affected by HCW engagement and financial incentives. Loyalty attitude itself has a direct impact on the respect of HCWs toward their direct managers and the perceived respect and trust of patients toward HCWs.

### 4.3. Strengths and limitations

Several strengths characterize this study. First, this is the first study to include HCWs in hospital evaluations based on BSC dimensions. Second, this is the first study to use the BSC-HCW1 survey to determine which experiences of HCWs predict their attitudes. This application will enable hospitals' executives to identify performance gaps based on the views and opinions of HCWs, which will ultimately help to improve Palestinian hospitals' PE. Third, this is the first study to examine the differences in experiences and attitudes between physicians and nurses in Palestinian hospitals. Fourth, this is one of the few research projects that tries to engage Palestinian HCWs in the PEs of Palestinian hospitals. Most existing research concentrated on gauging the satisfaction of HCWs and lacked distinguishing between their HCWs' experiences and attitudes. Consequently, this study will provide a greater comprehension of the predictors of these attitudes and the overall strategic map model of Palestinian hospitals from the HCW perspective. Fifth, to the best of our knowledge, this is the first study to investigate the PE of Palestinian hospitals during the pandemic period. In conclusion, this is one of the largest research initiatives that has ever been conducted with the participation of Palestinian hospitals. In this study, 30% of the Palestinian hospitals participated in the evaluation. In addition, we included all types of hospitals in our sample, including location, style of hospital management, hospital size, and accreditation status. In addition, the categories of patient HCWs varied according to their gender, age, profession, department, and region. This will enable the generalizability and comparability of the study's findings to other Palestinian hospitals and HCWs.

On the other hand, this research has some limitations. First, due to hospital permission restrictions, we did not include these factors in our statistical analysis for this research. After gaining authorization from eight hospitals to publish such an analysis, it is still necessary to conduct additional research to assess the impact of hospital and HCW features on HCW experiences and attitudes. Second, even though this instrument analyzes topics such as HCWs' knowledge updates on medications and diseases, it lacks COVID-19-specific questions, which is another drawback of this research. This refers to the reason this instrument was developed prior to the COVID-19 pandemic. Consequently, COVID-19-related elements might be evaluated in future versions of the BSC-HCW1 instrument. Third, several HCWs expressed reluctance to offer unfavorable comments on their managers' performance, which may have influenced the appraisal of this aspect. However, the researchers attempted to mitigate this bias by assuring all respondents both orally and in their written consent that their responses would remain anonymous and confidential and that only the final findings would be shared with their supervisors. Last, this study evaluates hospitals only from the perspective of HCWs. There is a need for research to assess these hospitals based on BSC perspectives and dimensions from other stakeholders, including management and patients, and to compare the evaluations altogether.

### 4.4. Practical and theoretical implications

This research offers broad practical implications for Palestinian hospital managers. To implement the third generation of BSCs in the future, hospital managers need to focus on designing targets, activities, and allocated budgets. Our recommendations for the practical implications of such action plans can be summarized as follows:

1. Reviewing the financial incentives system and linking it with HCWs' PE and achievements.
2. Training and coaching Palestinian HCWs on how to improve their workload time-life balance.
3. Investing in action plans on how to increase the time that Palestinian HCWs spend with their patients.
4. Planning and executing continuous educational programs to update Palestinian HCWs with information regarding diseases and medications related to their fields. Future utilization of mHealth for such purposes is recommended.
5. Performing a periodic evaluation of available equipment that requires maintenance or replacement. In addition, investments in electronic decision support systems can improve the quality and development factor.
6. Monitoring the PE of Palestinian HCWs on a quarterly basis and designing an appraisal system that explains to HCWs their strengths and weaknesses. In addition to communicating and discussing with them how to utilize their strengths and what actions or development programs are needed to improve their weaknesses.
7. Palestinian managers have to strengthen HCWs' engagement in planning and decision processes.
8. The managerial early awareness of the high-risk HCW groups who intend to leave their jobs and invest in improving their experiences encourages HCW loyalty attitudes, such as the improvement of HCWs' workload time-life balance, quality and development initiatives, managerial performance, HCW engagement and financial incentives.
9. Focusing on improving the factors that affect the respect of HCWs toward their direct manager, particularly HCW engagement, managerial performance, and HCWs' loyalty attitude.
10. Focusing on improving the factors that affect perceived patient respect toward HCWs, particularly quality and development initiatives, HCWs' workload time-life balance, loyalty attitudes, managerial performance, and financial incentives.

This study also has theoretical implications for future research:

1. Evaluating the effect of hospital and HCW characteristics on the experiences and attitudes of HCWs.
2. PE was compared based on the manager's evaluation and hospital records with the evaluations of other stakeholders, such as patients and HCWs.

## 5. Conclusion

In conclusion, in this research, it was possible to engage Palestinian HCWs in the assessment of Palestinian hospitals based on the BSC perspectives and dimensions. This study revealed that there are no differences between physicians and nurses regarding their evaluations. On the other hand, the HCWs' experiences that had the greatest positive impact on HCWs' loyalty attitudes were the HCWs' workload time-life balance, followed by the quality and development initiatives and managerial performance. HCW loyalty attitude was also positively affected indirectly by HCW engagement and financial incentives. The factors that directly affected the respect of HCWs toward their direct manager were HCW engagement, managerial performance, and HCW loyalty attitude. Managerial performance also had an indirect positive impact since quality and development initiatives worked as mediators. The factors that had the greatest direct impact on perceived patient respect toward HCWs were quality and development initiatives, followed by HCWs' workload time-life balance, loyalty attitudes, and managerial performance. Managerial performance and financial incentives also had an indirect effect on perceived patient respect. The technology perspective did not have a direct impact on improving HCWs' attitudes in general but had a role in predicting quality and development initiatives.

Despite the importance of these factors, their assessment revealed a great opportunity to improve. First, a consideration to improve financial motivation and link it with HCWs' PE and achievements must be considered. Second, more than half of the HCWs expressed their inability to spend sufficient time with the patients, which may have affected the quality and precision of their diagnosis and patient care. Third, almost 40% of the HCWs revealed that the hospitals do not provide them with education updates on medications or diseases. Education program development must be emphasized and included in hospitals' action plans. Fourth, ~40% of HCWs stated that the hospital equipment did not help them in offering high-quality services to patients. Continuous evaluation of which equipment requires maintenance or replacement is critical. Fifth, almost 35% of the HCWs revealed that their performance assessment does not explain their strengths and weaknesses. A lack of understanding weaknesses may hinder the opportunity for future improvements, and missing understanding of the strengths may lead to their underutilization and the opportunity for proper recognition and implicit motivations. Sixth, only half of the HCWs expressed that their managers engaged them in the planning and decision process. A greater emphasis on HCW engagement culture must be considered by Palestinian health managers. Seventh, only 60% of HCWs have loyalty attitudes, including their satisfaction and intent to stay. Managerial early awareness of high-risk groups and focusing on improving HCWs' loyalty attitudes will prevent the high turnover rates that come with avoidable recruitment and training costs and increased retention of valuable employees. These findings can be generalized to other Palestinian hospitals since this research was conducted at 30% of Palestinian hospitals and included all forms of hospital administration styles, all hospital sizes, and accreditation status in various locations.

## Data availability statement

The raw data supporting the conclusions of this article will be made available by the authors, without undue reservation.

## Ethics statement

The studies involving human participants were reviewed and approved by the Research and Ethics Committee at the Faculty of Medicine and Health Sciences at An-Najah National University with the reference code number (Mas, May/20/16) on 31 May 2020. The patients/participants provided their written informed consent to participate in this study.

## Author contributions

FA was responsible for planning this paper's conception, obtaining hospital approvals, data collection, statistical analysis, interpretation of data, and writing the final draft. AK, AA, IB, and DE substantially revised the final manuscript draft. FA, AK, AA, IB, and DE approved the submitted version, agreed to be personally accountable for the author's contributions, and to ensure that the accuracy and integrity of any part of the work were appropriately investigated and resolved. All authors contributed to the article and approved the submitted version.
